# Ciprofibrate therapy in patients with hypertriglyceridemia and low high density lipoprotein (HDL)-cholesterol: greater reduction of non-HDL cholesterol in subjects with excess body weight (The CIPROAMLAT study)

**DOI:** 10.1186/1475-2840-3-8

**Published:** 2004-07-23

**Authors:** Carlos A Aguilar-Salinas, Andréia Assis-Luores-Vale, Benjamín Stockins, Hector Mario Rengifo, José Dondici Filho, Abrahão Afiune Neto, Lísia Marcílio Rabelo, Kerginaldo Paulo Torres, José Egídio Paulo de Oliveira, Carlos Alberto Machado, Eliana Reyes, Victor Saavedra, Fernando Florenzano, Ma Victoria Hernández, Sergio Hernandez Jiménez, Erika Ramírez, Cuauhtémoc Vazquez, Saul Salinas, Ismael Hernández, Octavio Medel, Ricardo Moreno, Paula Lugo, Ricardo Alvarado, Roopa Mehta, Victor Gutierrez, Francisco J Gómez Pérez

**Affiliations:** 1 Departamento de Endocrinología y Metabolismo. Instituto Nacional de la Nutrición, México City, Mexico; 2 Hospital Socor. Dept° de Aterosclerose. Rua Tupis, 1540 – 1° andar – Barro Preto – Belo Horizonte/MG-30190-062, Brazil; 3 Universidad De La Frontera. Departamento Medicina Interna. Av. Francisco Salazar 01145 Temuco-Chile; 4 Centro Médico del Pacífico. Departamento de Endocrinología. Calle 5B No. 42-16. Cali – Colombia; 5 CINDI – Centro de Investigações Diagnósticas Ltda. Dept° de Cardiologia. Rua Rei Alberto, 196 – Centro – Juiz de Fora/MG – 36016-300, Brazil; 6 Hospital São Salvador. Dept° de Cardiologia. Avenida A, 333 – Setor Oeste – Goiânia/GO – 74110-020, Brazil; 7 Fundação Baiana de Cardiologia. Dept° de Lípides. Rua Augusto Viana, S/N° – Canela – Salvador/BA – 40140-060, Brazil; 8 Prócardio. Dept° de Aterosclerose. Avenida Nascimento de Castro, 1930 – Lagoa Nova – Natal/RN – 59056-450, Brazil; 9 Hospital Universitário Clementino Fraga Filho. Dept° de Diabetes/Nutrição. Avenida Brigadeiro Trompowiski, S/N° – Ilha do Fundão – Rio de Janeiro/RJ – 21941-590, Brazil; 10 Centro de Referência de Hipertensão Arterial Diabetes e Apoio à Saúde do Idoso Dept° de Centro de Referência em Dislipidemia Rua Doutor Clementino, 200 – Belém – São Paulo/SP – 03059-030, Brazil; 11 Hospital Dipreca. Unidad de Asistencia Nutricional Intensiva.División Medicina Interna Vital Apoquindo 1200. Las Condes.Santiago. Chile; 12 Consulta Privada. Guardia Vieja 181 of. 405. Providencia. Santiago-Chile; 13 Hospital Del Salvador. Sección Cardiología. Av. Salvador 364. Providencia. Santiago-Chile; 14 Hospital Fuerza Aérea De Chile. Unidad De Cardiología.Av. Las Condes 8631. Las Condes Santiago-Chile; 15 Hospital de Cardiología del Centro Médico Nacional Siglo XXI (IMSS).Depto. de Estudios Metabólicos y Clínica de Lípidos.Av. Cuauhtemoc No. 330 Col Doctores.México, D. F; 16 Hospital Juárez de México (SSA).Unidad Coronaria.Av. Instituto Politécnico Nacional 5160 Col Magdalena de las Salinas.México, D. F; 17 Hospital General Regional No. 72 (IMSS).Terapia Intensiva.Filiberto Gómez S/N y Vía Gustavo Baz.Tlalnepantla, Edo. de México, Mexico; 18 Centro Médico Nacional de Occidente (IMSS).Departamento de Cardiología.Belisario Domínguez sin número Col. Centro.Guadalajara, Jal, Mexico; 19 Hospital Dr. Santiago Ramón y Cajal (ISSSTE). Departamento de Cardiología Predio Canoa S/N Col. Los Angeles. Durango, Dgo, Mexico; 20 Asociación De Diabéticos De Chile. Quebec 496. Santiago-Chile

**Keywords:** Ciprofibrate, obesity, HDL cholesterol, triglycerides, fibrates

## Abstract

**Background:**

Hypertriglyceridemia in combination with low HDL cholesterol levels is a risk factor for cardiovascular disease. Our objective was to evaluate the efficacy of ciprofibrate for the treatment of this form of dyslipidemia and to identify factors associated with better treatment response.

**Methods:**

Multicenter, international, open-label study. Four hundred and thirty seven patients were included. The plasma lipid levels at inclusion were fasting triglyceride concentrations between 1.6–3.9 mM/l and HDL cholesterol ≤ 1.05 mM/l for women and ≤ 0.9 mM/l for men. The LDL cholesterol was below 4.2 mM/l. All patients received ciprofibrate 100 mg/d. Efficacy and safety parameters were assessed at baseline and at the end of the treatment. The primary efficacy parameter of the study was percentage change in triglycerides from baseline.

**Results:**

After 4 months, plasma triglyceride concentrations were decreased by 44% (p < 0.001). HDL cholesterol concentrations were increased by 10% (p < 0.001). Non-HDL cholesterol was decreased by 19%. A greater HDL cholesterol response was observed in lean patients (body mass index < 25 kg/m^2^) compared to the rest of the population (8.2 vs 19.7%, p < 0.001). In contrast, cases with excess body weight had a larger decrease in non-HDL cholesterol levels (-20.8 vs -10.8%, p < 0.001). There were no significant complications resulting from treatment with ciprofibrate.

**Conclusions:**

Ciprofibrate is efficacious for the correction of hypertriglyceridemia / low HDL cholesterol. A greater decrease in non-HDL cholesterol was found among cases with excess body weight. The mechanism of action of ciprofibrate may be influenced by the pathophysiology of the disorder being treated.

## Background

Hypertriglyceridemia in combination with abnormally low concentrations of HDL cholesterol (High Density Lipoprotein Cholesterol) is one of the most common and atherogenic profiles of lipid metabolism [1]. In the PROCAM study [2], the 6-year incidence of coronary events in men aged between 40 and 60 years, was twelve times higher than in the control group. The prevalence of this abnormality varies among ethnic groups [3]. It is found in 13% of the Mexican adults living in urban areas [4]. It is more common in men than in women (20.9% vs 7.2%) and in some age groups (i.e. men aged 30 to 39 years) this prevalence is as high as 30%. This lipid profile is the most frequent form of dyslipidemia in the metabolic syndrome [5]. However it is also found in subjects affected by primary dyslipidemias (e.g familial combined hyperlipidemia).

In the Veteran Affairs HDL Intervention Trial (VAHIT), the use of a fibrate (gemfibrozil) resulted in a 22% reduction in the incidence of cardiovascular events in subjects with low HDL cholesterol and a broad range of triglyceride values [6]. The benefit was accounted for by the positive effects obtained in cases with insulin resistance [7]. In spite of these positive results, there are few studies assessing the efficacy of other fibrates in the treatment of this form of dyslipidemia [8]. Relevant data such as the percentage of cases that achieve treatment goals are not described in the majority of these reports. Also, variables predicting a greater likelihood of achieving treatment goals remain to be identified. Our objective was to assess the efficacy and safety of ciprofibrate (100 mg/day) for the treatment of cases with hypertriglyceridemia / hypoalphalipoproteinemia in an open label, multicenter, international study. A clinically oriented approach is used for the description of the results.

## Materials and Methods

The trial included men and post-menopausal or non-pregnant women aged between 30 and 70 years who had hypertriglyceridemia (fasting concentrations between 1.68–3.9 mM/l (150 – 350 mg/dl) and hypoalphalipoproteinemia (HDL cholesterol ≤ 1.05 mM/l (40 mg/dl) for women and ≤ 0.92 mM/l (35 mg/dl) for men). The LDL cholesterol had to be lower than 4.2 mM/l (160 mg/dl). Patients were excluded if they had an acute coronary event during the three months preceding the study, type 1 diabetes, uncontrolled hypertension, severe renal dysfunction, nephrotic syndrome or aspartate aminotransferase (AST) or alanine aminotransferase (ALT) levels > 1.5 × the upper limit of normal (ULN), or if their creatine phosphokinase (CPK) levels were > 3 × ULN. Consumption of any lipid-altering drug within the previous 4 weeks (6 months for probucol) prevented entry into the study. Patients could be receiving other concomitant medication as long as the dosage was not modified during the study.

The Ethics Committee in each institution approved the protocol and every patient provided witnessed, written informed consent prior to entering the study.

This was a multicenter, international, open-label study. Patients were recruited from 25 lipid clinics from México (n = 152), Brazil (n = 129), Chile (n = 78) and Colombia (n = 78). Cases fulfilling the inclusion criteria were invited to participate. The initial visit included a medical evaluation, a physical examination and the prescription of an isocaloric diet consisting of 50% carbohydrate, 30% fat, 20% protein with a cholesterol content of 200 mg [9]. Blood samples were obtained after a 9–12 h fasting period. All patients were assigned to receive ciprofibrate 100 mg at bedtime. The second and final visit was scheduled 4 months later. During this visit drug compliance and safety profile were assessed and body weight as well as laboratory parameters were measured. Drug compliance was measured by counting the returned pills. Adherence to the diet was not assessed during the study

The primary efficacy parameter was the percentage change in triglycerides from baseline. Secondary efficacy parameters included the percentage change in total cholesterol, HDL cholesterol and non-HDL cholesterol from baseline. Non-HDL cholesterol was calculated by subtracting the HDL cholesterol from the total cholesterol levels. In a post hoc analysis, the percentage of cases that achieved the treatment goals proposed by the ATP-III recommendations [10] on the final visit was also estimated.

At each visit, AST, ALT, fasting plasma glucose and CPK levels were measured. Clinically relevant complications were defined as either CPK > 5 × ULN accompanied by muscle pain, tenderness or weakness or ALT or AST > 3 × ULN. Patients were excluded from the study if they developed severe hyperglycemia or any other significant complication to treatment. Other reasons for premature withdrawal were lack of compliance to the medication. Cases were instructed to contact their physician in case of any side effect.

All samples were analyzed in a central laboratory (Quest laboratories). In Brazil, the local laboratory of every center was used instead. Blood samples were taken after an overnight fast (≥ 9 hours). Measurements were performed during the first 24 hours after the blood was drawn; blood samples were kept at 4°C until the analysis. All laboratory analyses were performed with commercially available standardized methods. Glucose was measured using the glucose oxidase method. Total serum cholesterol and triglycerides levels were measured using an enzymatic method. HDL cholesterol levels were assessed using phosphotungstic acid and Mg2+.

Statistical analysis was performed using SPSS for Windows version 10. An intention to treat analysis was used. Two sided ANOVA tests were used for assessing differences between groups for continuous variables. All categorical variables were analyzed using the chi squared test. Multiple logistic regression models were constructed for the identification of variables associated with the achievement of treatment goals.

## Results

Four hundred thirty seven patients were included. The clinical characteristics of the study subjects are shown in table 1. Almost half of the population had a body mass index between 25 and 30 kg/m^2 ^(n = 221); an additional 32.5% were obese (n = 142) Diabetes was present in 125 subjects (28.7%).

**Table 1:** Baseline characteristics of the patients included in study (n = 437)

**Variable**	**N = 437**
Sex	
Male (n(%))	239 (54.7)
Female (n(%))	198 (45.3)
Age (years)*	54.7 ± 12.1
Body mass index (kg/m^2^)	28.8 ± 4.6
Diabetes (n(%))	125 (28.7)
Fasting plasma glucose (mM/l)*	5.2 ± 0.6
Family history of dyslipidemia (n(%))	171 (39.2)
Coronary heart disease (n(%))	196 (45)
Aspartate aminotranferase (mU/l)	23 ± 11
Alanine aminotransferase (mU/l)	26 ± 14

Data expressed as mean ± standard deviation. *To convert to mg/dl, multiply by 18

Both evaluations were completed in every case. The medication was stopped before the trial was completed by 117 subjects (26.7%). In the majority of cases this was not related to side effects (see below). In addition, 46 cases (10.5%) had poor compliance to the medication. The body weight remained constant in all patients. The alcohol and tobacco consumption was not modified during the study.

Ciprofibrate treatment and diet had a significant beneficial effect on the lipid profile, as shown in table 2. After 4 months of treatment, plasma triglyceride concentrations were decreased by 44% (p < 0.001). HDL cholesterol concentrations were increased by 10.1% (p < 0.001). Non-HDL cholesterol was decreased by 19.2% (p < 0.001). Total cholesterol was also favorably modified (-14.9%, p < 0.001). In contrast, LDL cholesterol had a minor modification (-5.4%, p < 0.001). A significant decrease in fasting glycemia was observed in both obese and diabetic cases. This change was not found in lean subjects.

**Table 2:** Changes in the lipid profile and clinical characteristics between baseline and post-treatment values

	**Intention to treat analysis N = 437**		
	**Baseline**	**Final**	**Percent change**
Triglycerides (mM/l) ^†^	3.01 ± 0.7	1.61 ± 0.8	-44 ± 33*
HDL Cholesterol (mM/l)^‡^	0.91 ± 0.1	0.98 ± 0.4	10 ± 52*
Non-HDL cholesterol (mM/l)^‡^	4.57 ± 0.9	3.61 ± 1.5	-19 ± 36*
Cholesterol (mM/l)^‡^	5.5 ± 0.9	4.57 ± 1.8	-14.9 ± 35*
LDL Cholesterol (mM/l)^‡^	3.1 ± 0.9	2.8 ± 1.3	-5.4 ± 59*

Mean ± standard deviation are presented. *p < 0.001 ^†^To convert to mg/dl multiple by 89 ^‡^To convert to mg/dl multiple by 38

The achievement of treatment goals is the ultimate aim of lipid-lowering therapy. Almost half of the cases had reduced their triglyceride concentrations below 1.68 mM/l (150 mg/dl) (n= 191(43.7%). HDL cholesterol levels above 1.05 mM/l (40 mg/dl) were found in 51% of the cases (n = 223). Also, a significant proportion of the subjects (63.15%, n = 276) achieved the non-HDL cholesterol goal 4.2 mM/l (< 160 mg/dl). The LDL cholesterol goal < 3.4 mM/l (< 130 mg/dl) was attained by 56.2 % (n = 246). A full correction of the hypertrigliceridemia / low HDL cholesterol occurred in 129 subjects (29.5%). Many of them also had a non-HDL cholesterol level below 4.2 mM/l (160 mg/dl) (n = 101, 23.1%)

**Figure 1:**
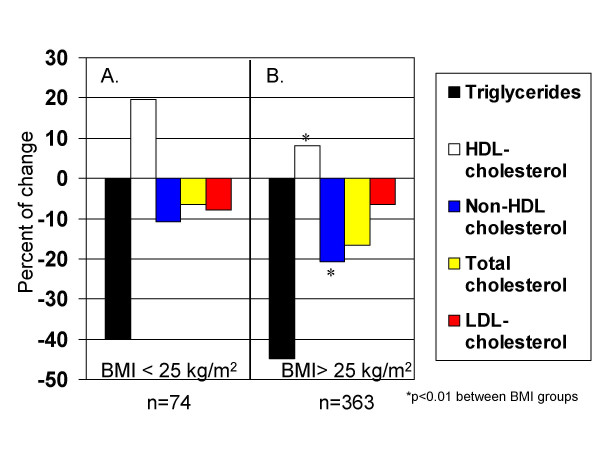
The percentage of change in the lipid parameters is different in cases with a body mass index above 25 kg/m^2 ^compared to the response observed in lean individuals during treatment with ciprofibrate and diet.

The lipid response during treatment differed between cases with a body mass index above or below 25 kg/m^2^. As is shown in figure 1, the percentage change in HDL cholesterol was higher in lean subjects. In contrast, the non-HDL cholesterol concentration had a significantly greater reduction among subjects with excess body weight. Both differences remained significant even after adjusting for age and gender. The lipid profile did not differ between these groups during the initial visit.

Patients with diabetes had moderate hyperglycemia during the study. Their fasting glycemia was 7.7 ± 2.6 mM/l (139 ± 47 mg/dl) at baseline. A small but statistically significant decrease in glucose concentration was observed at the end of the trial (5.7 ± 3 mM/l, 104 ± 54 mg/dl p < 0.001). At baseline, their lipid profile differed, from the non-diabetic subjects, only with regards to higher triglyceride concentrations (3.13 ± 0.97 vs 2.95 ± 0.66 mM/l, p < 0.001). The lipid response to ciprofibrate and dietary treatment did not differ from that observed in the whole group. Individuals with a family history of dyslipidemia (n = 171) had higher cholesterol, triglycerides and LDL cholesterol at baseline compared to the rest of the population. The lipid response to treatment was similar to that reported in the whole group.

Multiple regression models were constructed to identify variables associated with the achievement of the treatment goals. For every target value, the main determinants were the baseline value and the percentage change after treatment. No other parameter provided additional information in any of the models. Thus, we analyzed the variables associated with the percentage change during treatment. The non-HDL cholesterol model provided more information compared to models derived from the other lipid parameters. In the non-HDL cholesterol model, a body mass index greater than 25 kg/m^2^, the triglyceride response and the coexistence of cholesterol above 5.2 mM/l (200 mg/dl) (mixed hyperlipidemia) were predictors for a greater non-HDL cholesterol response (table 3). For both, the HDL cholesterol and the triglycerides models, the inclusion of a body mass index < 25 kg/m^2 ^added little information and had only borderline statistical significance. No other clinically relevant variable was associated with the percentage change of any other lipid parameter.

**Table 3:** Multiple regression model using as dependent variable percent of change in non-HDL cholesterol concentration

**Variable**	**Beta coefficient ± standard error**	**p value**
Constant	14.4 ± 2.6	< 0.001
BMI < 25 kg/m^2^	7.6 ± 3.6	0.036
Mixed hyperlipidemia	0.82 ± 0.2	0.003
Percent of change of triglycerides	0.68 ± 0.4	< 0.001

Correlation coefficient = 0.647, r^2 ^= 0.419, p = 0.001 BMI, Body mass index

Liver function tests were not modified by the treatment. No patient had a significant alteration of any of the laboratory tests. There were no incidents of either myopathy or liver dysfunction. No persistent elevations in ALT, AST or CPK, defined as clinically relevant, were reported during the course of the study.

## Discussion

The combination of hypertriglyceridemia / low HDL cholesterol is a common abnormality of lipoprotein metabolism and is associated with increased cardiovascular risk. Our data show that ciprofibrate and an isocaloric diet are an effective treatment for this dyslipidemia. However, there was significant variation in response to treatment between individuals. Excess body weight may be an important determinant of the lipid response. It is associated with a greater degree of non-HDL cholesterol reduction and a relatively smaller elevation in HDL cholesterol.

A significant improvement in plasma triglycerides and HDL cholesterol concentrations resulted from the administration of ciprofibrate and dietary modifications. Our results are in agreement with previous studies [11-15]. This study differs from previous reports due to its design. We wanted to assess the lipid response to ciprofibrate in a real life environment. Hence, the highly controlled conditions of a randomized, double blind study were avoided. Also, we limited our inclusion criteria to subjects with hypertriglyceridemia / low HDL cholesterol, instead of including subjects with a wide variety of lipid profiles. The results are in accordance with the uncontrolled design of the study. By the use of ciprofibrate and isocaloric diet, almost half of the cases achieved the triglyceride goal (1.68 mM/l, 150 mg/dl). HDL cholesterol levels above 40 mg/dl were found in 51% of the cases. The full correction of the combination of hypertriglyceridemia / low HDL cholesterol occurred in a third of the population. This rate is similar to that reported for the LDL cholesterol goals achieved by the use of statins. Thus, our results reflect the strengths and limitations of treating this lipid abnormality in an uncontrolled setting.

A large range of lipid responses was observed between individuals. There are few reports designed to analyse the determinants of the lipid response to fibrates. Robins reported a lower HDL cholesterol elevation with a fibrate when insulin resistance is present [7]. In this report, a lower HDL cholesterol response was observed in patients with excess body weight (body mass index above 25 kg/m^2^) in comparison to that found in lean individuals. Since excess body weight is strongly associated with insulin resistance [16,17], our observations may be in agreement with the findings of Robins. Interestingly, the presence of insulin resistance was associated with the lowest incidence of coronary events in the VAHIT study. In our report, obese individual had a larger decrease in non-HDL cholesterol levels. The greater response in non-HDL cholesterol observed in our obese (and possibly insulin resistant) subjects may be one possible explanation for the greater benefit found in insulin resistant subjects during the VAHIT study. Our data suggest that the mechanism of action of ciprofibrate may be altered by the pathophysiology of the disorder being treated. The same phenomenon has been observed with the use of statins [18,19]. The greater reduction in non-HDL cholesterol in subjects with excess body weight could be explained by an increased clearance of remnants, IDL and VLDL particles. Ciprofibrate may enhance their clearance either by decreasing the concentration of the apolipoprotein CIII (an inhibitor of the lipolytic activity of the lipoprotein lipase) [20-22] or by increasing the mass and activity of lipoprotein lipase [23]. Genes encoding apolipoprotein CIII and lipoprotein lipase contain a PPAR-alpha response element; hence their expression may be modified during treatment with a fibrate [24]. Additional studies are needed to identify other possible determinants of the lipid response to treatment with a fibrate.

Several limitations of the study must be recognized. The uncontrolled design resulted in a relatively high rate of drug discontinuation. However, this phenomenon is a common finding in studies done in open populations assessing the adherence to different lipid-lowering medications [25]. To overcome this limitation, an intention to treat analysis was used. Also, the lack of a run-in period in which the effect of diet could be measured and the absence of information about the adherence to the diet prevented us from discerning to what extent the observed result was due to fibrate alone. Finally, some of the conclusions, like the identification of the determinants of the lipid response, came from a post hoc analysis.

In conclusion, ciprofibrate is effective in the treatment of patients with hypertriglyceridemia / low HDL cholesterol. Significant reductions in triglycerides and non-HDL cholesterol resulted from ciprofibrate therapy. In addition, higher HDL cholesterol levels were found at the end of the treatment. Excess body weight alters the lipid response to ciprofibrate. A greater non-HDL cholesterol lowering is achieved in subjects with excess body weight compared to that found in lean individuals. Controlled trials are needed to compare the lipid-lowering effects of ciprofibrate in groups of subjects defined by their adiposity or other markers of insulin resistance.

## Competing interests

This study was supported by an educational grant provided by Sanofi-Synthelabo. It included the study expenses and it will cover the article processing charge. No other competing interest need to be declared

## Author contributions

CAAS participated in the design of the study, performed the statistical analysis and drafted the manuscript. AALV participated in the design of the study and in the preparation of the manuscript. FJGP participated in the design of the study and in the preparation of the manuscript. All other authors were responsible of the inclusion and follow-up of the study subjects. All authors read and approved the final manuscript.
